# Structure and absolute configuration of natural fungal product beauveriolide I, isolated from *Cordyceps javanica*, determined by 3D electron diffraction

**DOI:** 10.1107/S2053229624001359

**Published:** 2024-02-27

**Authors:** Kshitij Gurung, Petr Šimek, Alexandr Jegorov, Lukáš Palatinus

**Affiliations:** a Department of Structure Analysis, Institute of Physics of the Czech Academy of Sciences, Na Slovance 1999/2, Prague 8, 18221, Czech Republic; b Biology Centre, Czech Academy of Sciences, Branišovská 1160/31, České Budějovice 2, 370 05, Czech Republic

**Keywords:** crystal structure, natural product, 3D electron diffraction, absolute structure, Alzheimer’s disease

## Abstract

Beauveriolides, including the main beauveriolide I {systematic name: (3*R*,6*S*,9*S*,13*S*)-9-benzyl-13-[(2*S*)-hexan-2-yl]-6-methyl-3-(2-methyl­prop­yl)-1-oxa-4,7,10-tri­aza­cyclo­tridecane-2,5,8,11-tetrone, C_27_H_41_N_3_O_5_}, are a series of cyclo­depsipeptides that have shown promising results in the treatment of Alzheimer’s disease and in the prevention of foam cell formation in atherosclerosis. Their crystal structure studies have been difficult due to their tiny crystal size and fibre-like morphology, until now. Recent developments in 3D electron diffraction methodology have made it possible to accurately study the crystal structures of submicron crystals by overcoming the problems of beam sensitivity and dynamical scattering. In this study, the absolute structure of beauveriolide I was determined by 3D electron diffraction. The cyclo­dep­si­peptide crystallizes in the space group *I*2 with lattice parameters *a* = 40.2744 (4), *b* = 5.0976 (5), *c* = 27.698 (4) Å and β = 105.729 (6)°. After dynamical refinement, its absolute structure was determined by comparing the *R* factors and calculating the z-scores of the two possible enanti­omorphs of beauveriolide I.

## Abstract 2

The crystal structure of the natural fungal product beauveriolide I, isolated from the fungus *Cordyceps javanica* (Bally) Kepler, B. Shrestha & Spatafora, was subjected to 3D electron diffraction experiment and its absolute structure was determined by dynamical refinement.

## Introduction

**Figure 1:**
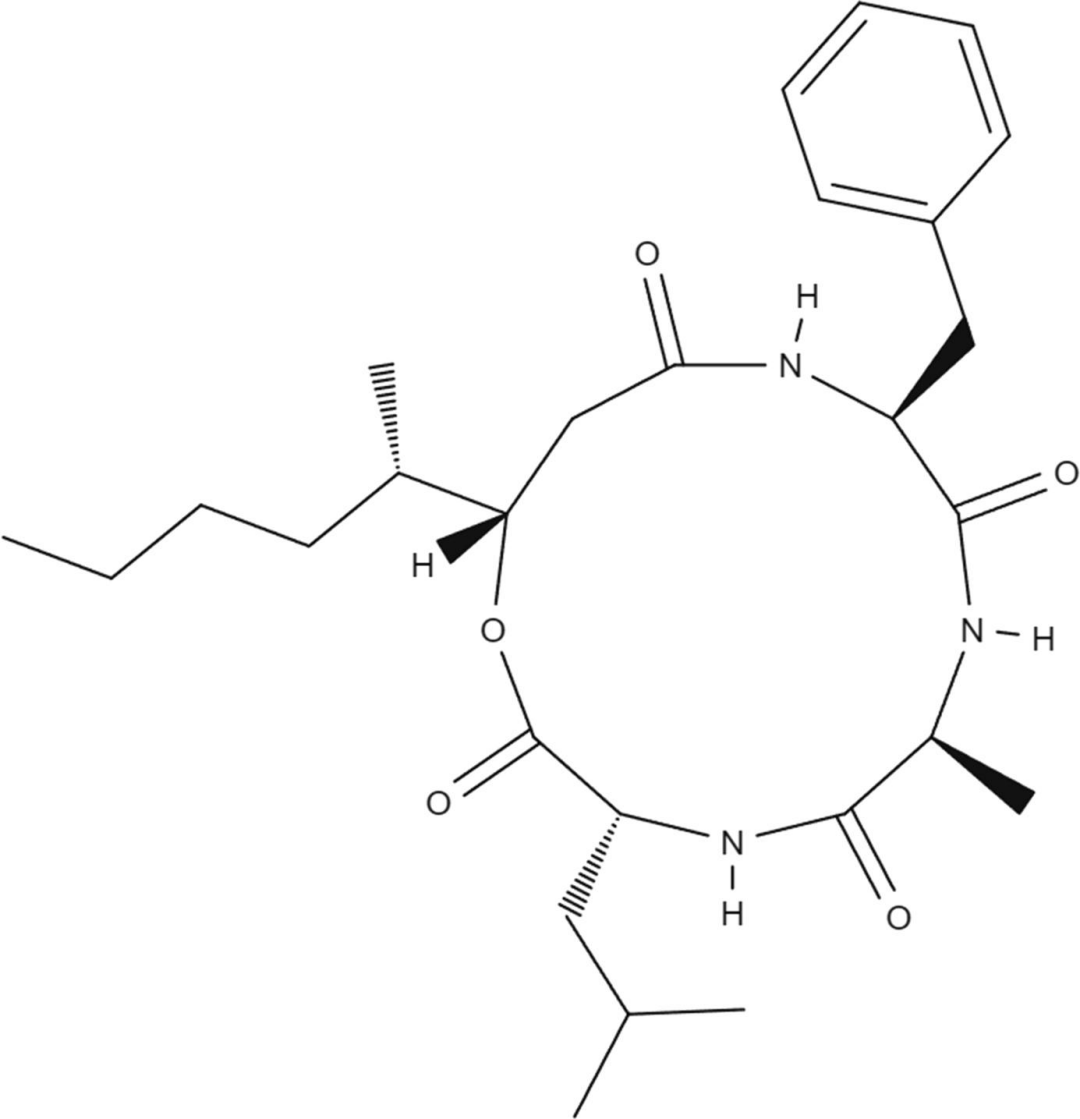
The mol­ecular structure of beauveriolide I.

Beauveriolides represent a series of cyclo­depsipeptides containing three amino acids and the unusual (3*S*,4*S*)-hy­droxy-4-methyl­hydroxy acid. They were first described as beauveriolide metabolites of the entomopathogenic fungus *Beauveria bassiana* (Elsworth & Grove, 1977 ▸). Similar metabolites were further described in several other fungi of the genera Beauveria, Isaria or Paecilomyces (Kadlec *et al.*, 1994 ▸). The absolute configuration of the (3*S*,4*S*)-hy­droxy acid component of beauveriolide I and II isolated from various Beauveria species was first estimated by synthesizing all possible chiral variants and comparing their ^1^H and ^13^C NMR spectra and optical rotation data (Mochizuki *et al.*, 1993 ▸). Recently, the beauveriolides (beauverolides) have attracted attention as potential drugs for the treatment of Alzheimer’s disease and for preventing foam cell formation in atherosclerosis (Nagai *et al.*, 2008 ▸; Heneberg *et al.*, 2020 ▸). In particular, beauveriolide I (Fig. 1 ▸) has previously shown potent activity in inhibiting the formation of lipid droplets in mouse macrophages by specifically inhibiting the activity of acyl-coenzyme A (CoA):cholesterol acyl­transferase (ACAT) (Namatame *et al.*, 2004 ▸; Tomoda & Doi, 2008 ▸). Inhibition of ACAT also reduces the secretion of amyloid-β peptide (Huttunen *et al.*, 2007 ▸; Puglielli *et al.*, 2001 ▸), the accumulation of which in brain *loci* is known to progress Alzheimer’s disease (Hardy & Selkoe, 2002 ▸).

Beauveriolides form very small fibre-like crystals. Therefore, the determination of their single-crystal structure has never been successful, which, together with the difficulty of correctly identifying 3-hy­droxy-4-methyl­hydroxy acid and its chirality led to probably identical metabolites from several fungi being described under different names. This ambiguity still exists today. The structure and conformation of beauveriolides remain important for understanding their physical properties, their role in the self–nonself recognition as fungal metabolites by the insect immune system, and for investigating their potential role in the treatment of human diseases.

In the last decade, thanks to the tremendous progress in data acquisition and processing, 3D electron diffraction (ED) has become an effective tool in crystallography for determining the structure of crystals of various compounds, including inorganics, organics, metal–organic frameworks (MOFS) and biological samples. The great advantage of 3D ED is that in a transmission electron microscope (TEM), very small crystals with a volume in the range of 10^0^ to 10^−5^ µm^3^ can be easily located, and the beam can be focused to perform ED measurements (Gemmi *et al.*, 2019 ▸). Continuous rotation electron diffraction (cRED) has become the most common data-acquisition technique in 3D ED, where a sample is continuously rotated over a range, while the diffraction data is collected at a certain tilt-step. Thus, cRED enables fast data collection, minimizing the electron dose on the sample, and making it very useful for beam-sensitive samples, including organic samples. The electrons used in 3D ED inter­act much more strongly than the more commonly used X-ray, resulting in multiple scattering of the beam, called dynamic scattering, and described by the dynamical theory of diffraction. The dynamical diffraction causes nonlinear deviations in the diffracted intensities from the kinematic limit. A technique called dynamical refinement (Palatinus *et al.*, 2015 ▸) takes these effects into account in the calculation of model intensities during structure refinement, giving more accurate and reliable results than those obtained without the application of the dy­namical diffraction theory. In addition, the dynamical effects are sensitive to the absolute structure of non­centro­symmetric crystals (Spence *et al.*, 1994 ▸), enabling accurate structure determination and structure configuration of the crystals (Klar *et al.*, 2023 ▸; Brázda *et al.*, 2019 ▸).

In this study, we collected the 3D ED patterns of beauveriolide I using cRED experiments to solve its crystal structure. Despite the difficulties with the data quality resulting from the beam sensitivity of the crystal, we obtained satisfactory dynamical refinement, including the determination of absolute structure, thus confirming the absolute configuration of the chiral centres of beauveriolide I.

## Experimental

### Isolation of beauveriolide I

The surface stationary cultivation of *Cordyceps javanica* CCM8917 was carried out on a medium containing glucose (40 g), sorbitol (20 g), mannitol (10 g), soya peptone (30 g), KH_2_PO_4_ (1 g), MgSO_4_·7H_2_O (0.1 g), ZnSO_4_·7H_2_O (0.01 g) and water (1 l), for 18 d at 297 K. The isolated mycelium was washed with water and extracted several times with methanol (2 l). The extract was evaporated to dryness on a vacuum evaporator. HPLC–MS analysis revealed that the major isolated cyclo­depsipeptide had characteristics of previously described beauveriolide I (Mochizuki *et al.*, 1993 ▸); the other beauveriolides were M, F, L and Q in an approximate ratio of 40:20:20:20 (% with respect to beauveriolide I as 100%). The crude mixture was purified first by column chromatography on silica gel with a stepwise gradient of di­chloro­methane/methanol. The final purification was carried out by preparative HPLC chromatography using a 354 mm × 18 mm inter­nal diameter column, with Luna C8, 10 µm, and isocratic elution with methanol (85%) and water (15%). The crystalline material was obtained by the stepwise addition of water to the methano­lic solution of beauveriolide I. Finally, the crystals were isolated by filtration and dried in air.

### 3D electron diffraction (ED) experiment

The white powdery beauveriolide I sample was gently ground in an agate mortar. A TEM copper grid with holey carbon film was gently slid on the sample to stick some of the crystals onto the grid, and the excess was gently tapped off. The grid was loaded onto a cryo-holder and was inserted into an FEI Tecnai G2 20 TEM. The holder was then cooled to a temperature of 100 K before performing any measurements. The microscope was operated at 200 kV with a LaB_6_ cathode, equipped with a Medipix 3 hybrid pixel detector ASI Cheetah (512 × 512 pixels, 24-bit dynamic range). The tilt step per frame was 0.3°, with the exposure time ranging from 504 to 1014 ms per frame. The crystals were found to be too sensitive to the electron beam to permit the collection of a full data set on a single crystal. Therefore, data sets from four different crystals, labelled **a**–**d**, were merged to obtain a complete data set for the structure solution.

### Data reduction and refinement

**Figure 2:**
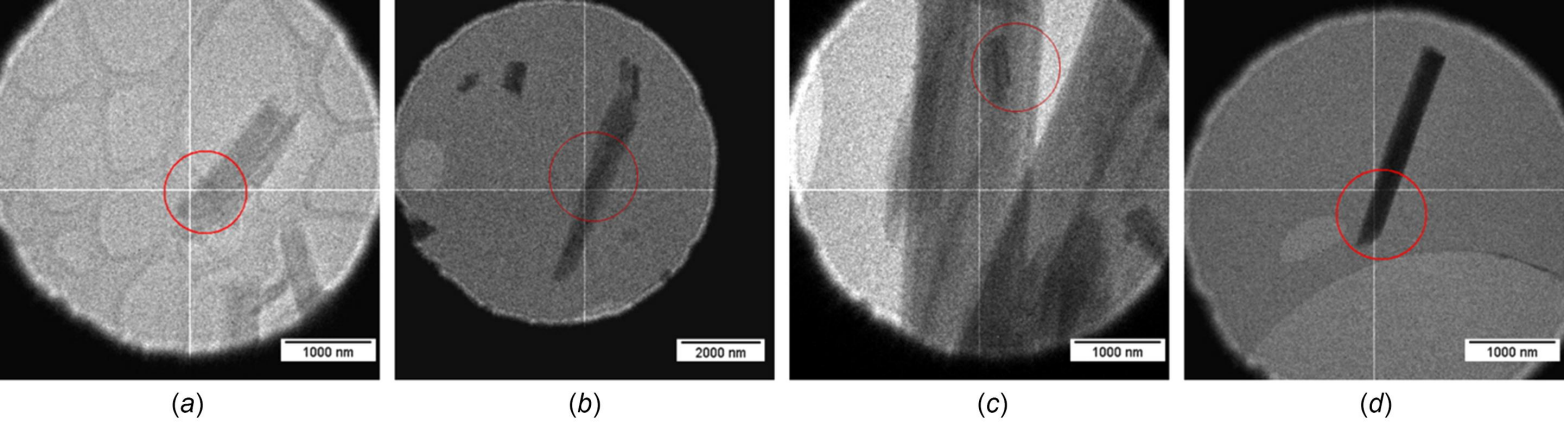
Crystals of beauveriolide I used for the 3D ED measurements. The red circles indicate the size of the illuminating electron beam.

Indexation, lattice parameter determination and peak integration were performed using *PETS2* (Palatinus *et al.*, 2019 ▸). The processed data were imported into *JANA2020* (Petříček *et al.*, 2023 ▸) and the crystal structure was solved using *SHELXT* (Sheldrick, 2015 ▸). All non-H atoms were found in the solution. For the refinement, only crystals **b** and **c**, shown in Fig. 2 ▸, were used since the data from the other two crystals were very weak and yielded poor refinement *R* factors.

Detailed 3D ED set-up, crystal information and refinement details are given in Table 1 ▸.

## Results and discussion

### Structure solution and refinement

The crystal lattice of beauveriolide I was determined to be a body-centred monoclinic lattice with lattice parameters of *a* = 40.2744 (4), *b* = 5.0976 (5), *c* = 27.698 (4) Å and β = 105.729 (6)°. The reflection condition *h* + *k* + l = 2*n* in the 2D reconstruction of reciprocal sections (Fig. S1 in the supporting information) indicates an *I*2 space group, which was further confirmed by the successful structure solution and refinement. There are two independent mol­ecules in the asymmetric unit. The standard setting *C*2 was not used, because it leads to a very high monoclinic angle of 140.87°. Fig. S2 in the sup­porting information shows the structure of beauveriolide I down the *c* axis.

**Figure 3:**
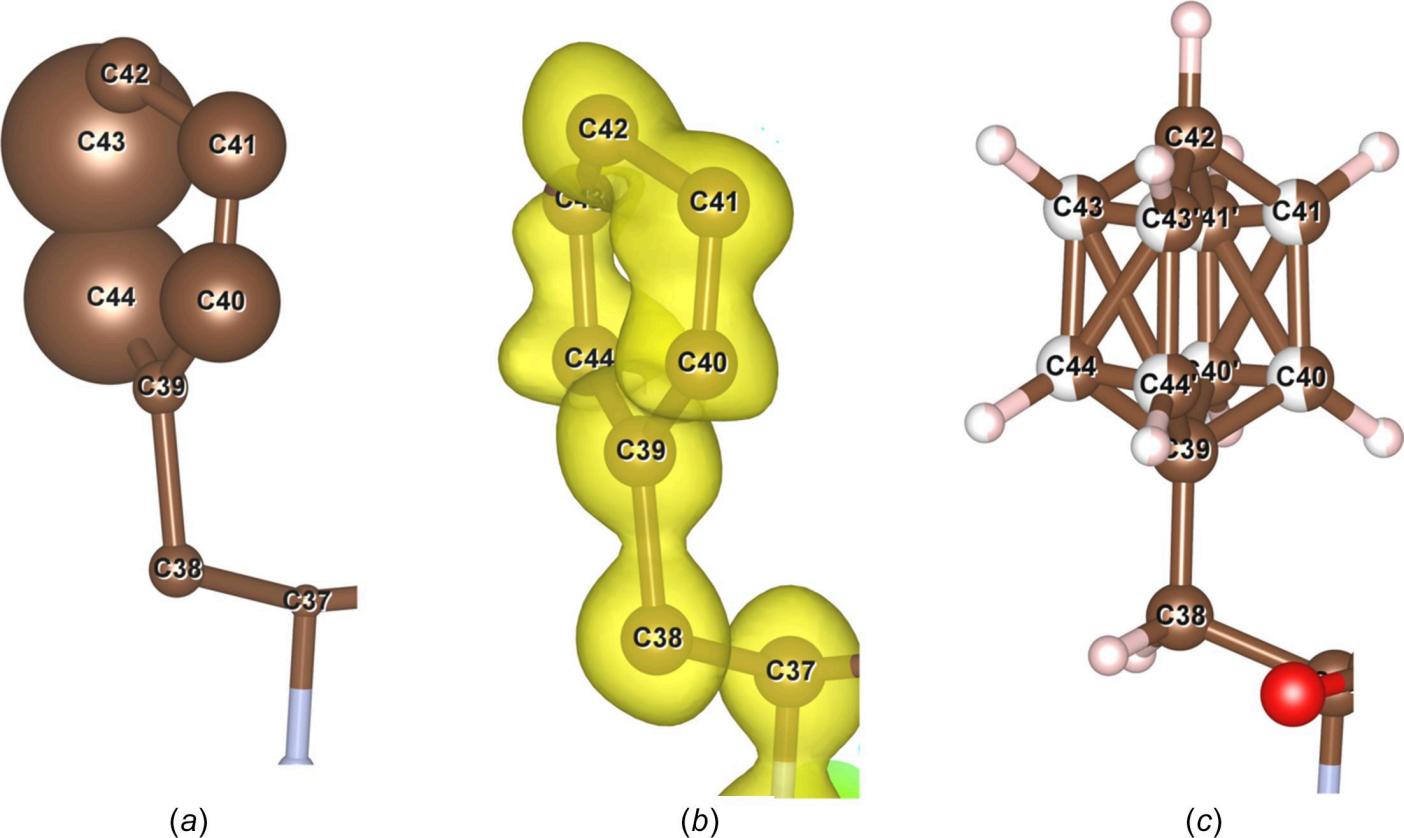
The benzene ring of one of the two asymmetric units of the solved beauveriolide I structure after an initial kinematical refinement, showing its distorted shape, together with (*a*) very high ADPs. (*b*) Fourier map showing four of the C atoms (C40, C41, C43 and C44) to be disordered. (*c*) The positions of the four C atoms and the bonded H atoms were split into two positions.

It can be observed clearly in Fig. 3 ▸(*a*) that one of the two mol­ecules in an asymmetric unit has a distorted benzene ring with very large atomic displacement parameters (ADPs) for four C atoms (labelled C40, C41, C43 and C44 in Fig. 3 ▸). The electrostatic potential map after an initial dynamical refinement [Fig. 3 ▸(*b*)] shows the broadening of the potential around the C atoms. Therefore, to better model this benzene ring, the four C atoms along with their H atoms were split equally into two different positions [Fig. 3 ▸(*c*)]. The relative occupancy of the two positions was refined freely. Subsequent refinements led to significantly improved ADPs of these C atoms, as well as improved *R* factors.

**Figure 4:**
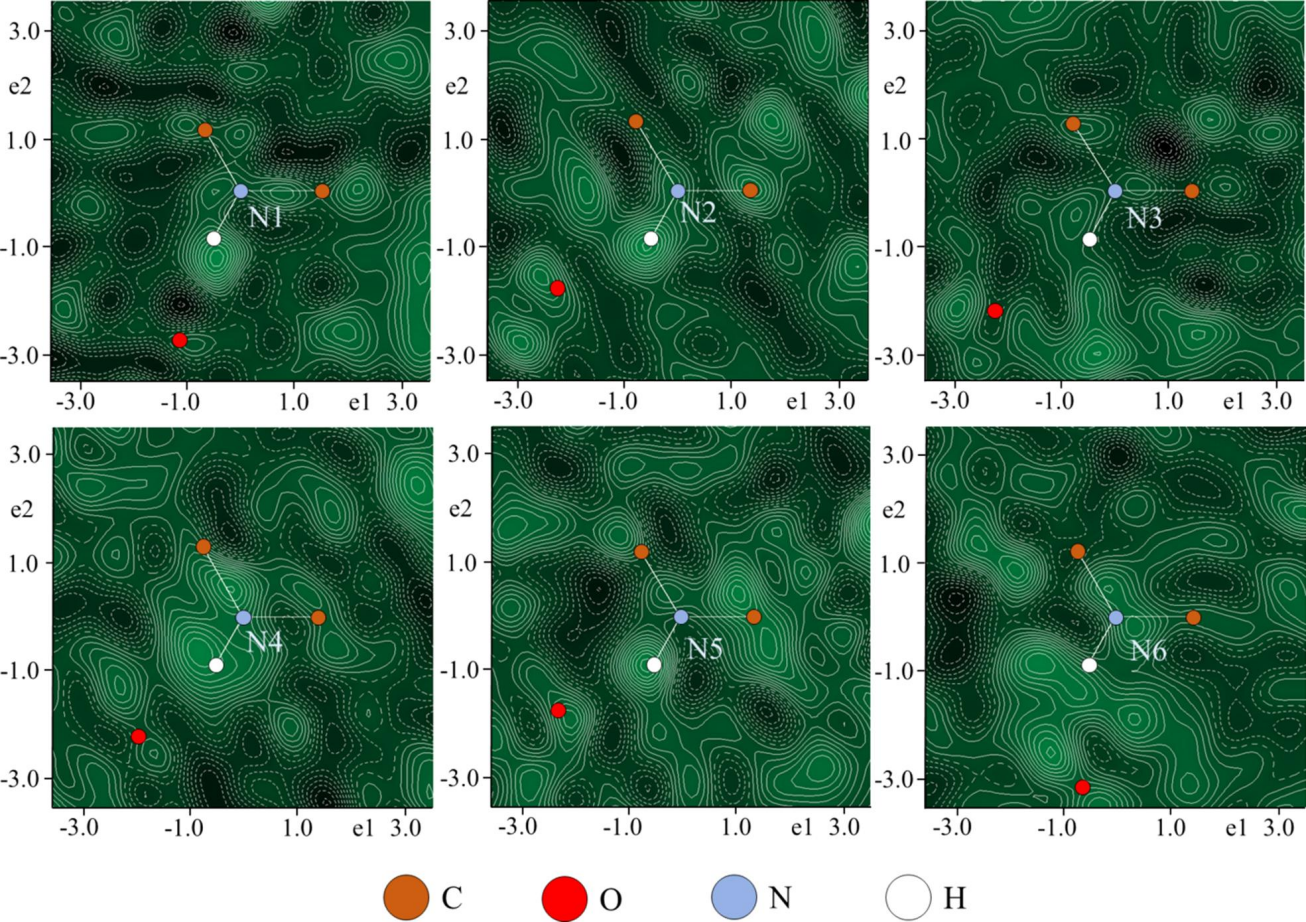
DESP maps in the C—N(H)—C planes of the two asymmetric units of the beauveriolide I structure. The black and green colours in the maps represent negative and positive contours, respectively, with a cutoff range of −0.561 to 0.751 e Å^−1^. The H atoms displayed are at their expected positions.

The H atoms for all the C atoms were added to geometrically determined positions. These H atoms were refined with *U*_iso_(H) = 1.2*U*_iso_(C). The C—H distances were fixed to 1.06 Å, *i.e.* to the inter­nuclear distances, as electron diffraction does not suffer from the biased H-atom positions in the same way as X-ray diffraction data. To determine the arrangement of the H atoms bonded to the N atoms, initial dynamical refinement of the crystal structure was performed without the H atoms, and the difference electrostatic potential (DESP) map was calculated to attempt the localization of the H-atom positions (Fig. 4 ▸). The H-atom positions shown in Fig. 4 ▸ are in their expected positions that form trigonal planar geometry with the N atoms and the two adjacent C atoms. Of the six N atoms in the asymmetric unit, the H atoms of four of them (N1, N2, N4 and N5) are clearly visible, with a density maximum between the N atoms and their adjacent O atoms. The lack of visibility of the H atoms in the other two N atoms is likely due to the data quality, which in turn, is likely due to the electron-beam sensitivity of the samples. For the final refinement, each of the six H atoms on the amine N atoms was added and fixed in the geometrically expected positions, with N—H distances of 1.01 Å and *U*_iso_(H) = 1.2*U*_iso_(N).

**Figure 5:**
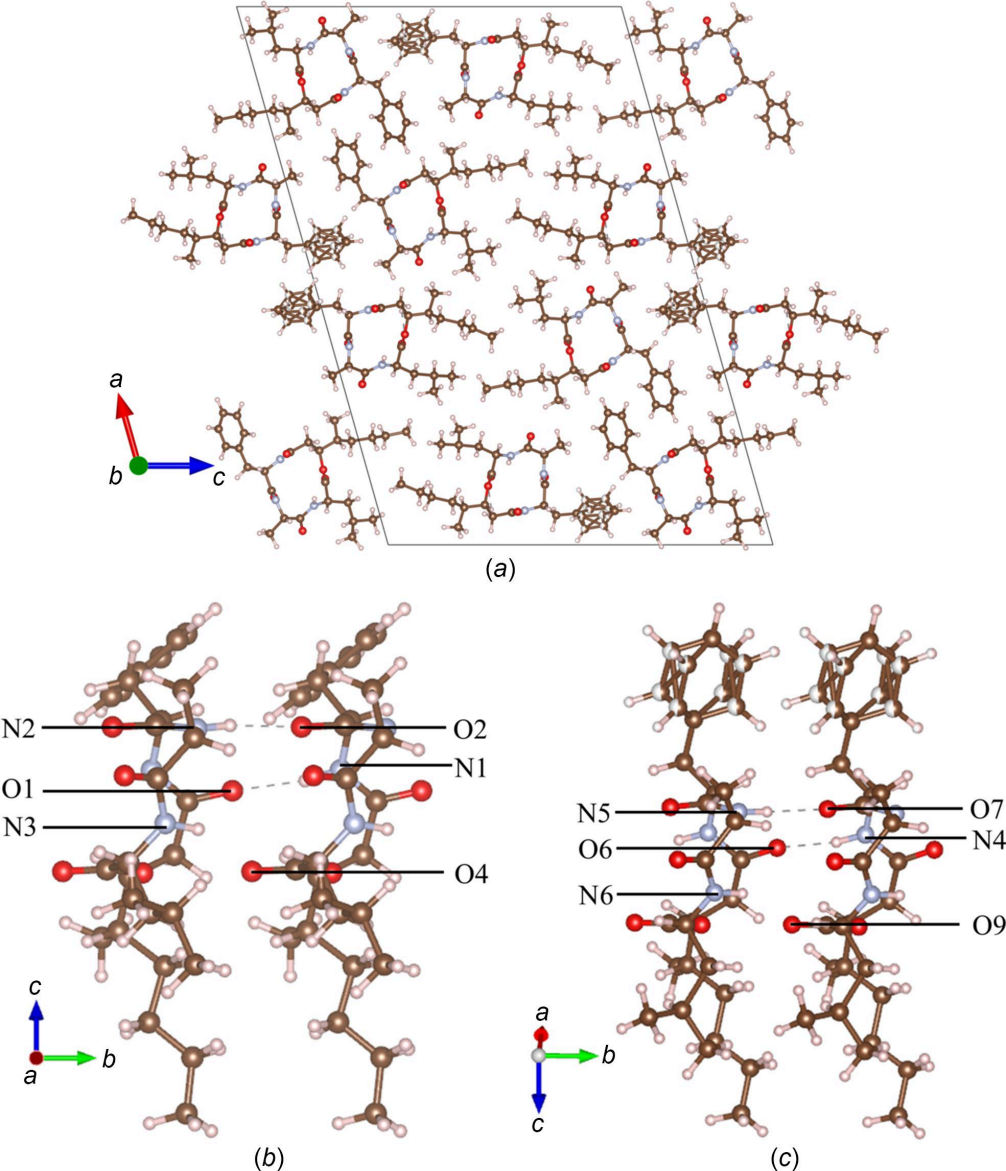
(*a*) A view of the crystal structure of beauveriolide I along the *b* axis after final dynamical refinement. (*b*)/(*c*) Hydrogen bonds stabilizing the stacking of the mol­ecules along the *b* axis.

The final refined structure of beauveriolide I [Fig. 5 ▸(*a*)] shows the stacking of the same asymmetric units along the *b* axis. As can be seen in Figs. 5 ▸(*b*) and 5(*c*), the stacking is stabilized by three hydrogen bonds between two mol­ecules. Regarding the distorted benzene ring, the split model considerably decreases the ADPs of the atoms. The split was found to be almost even, with an occupancy ratio of 0.47:0.53.

### Determination of absolute configuration and absolute structure

**Figure 6:**
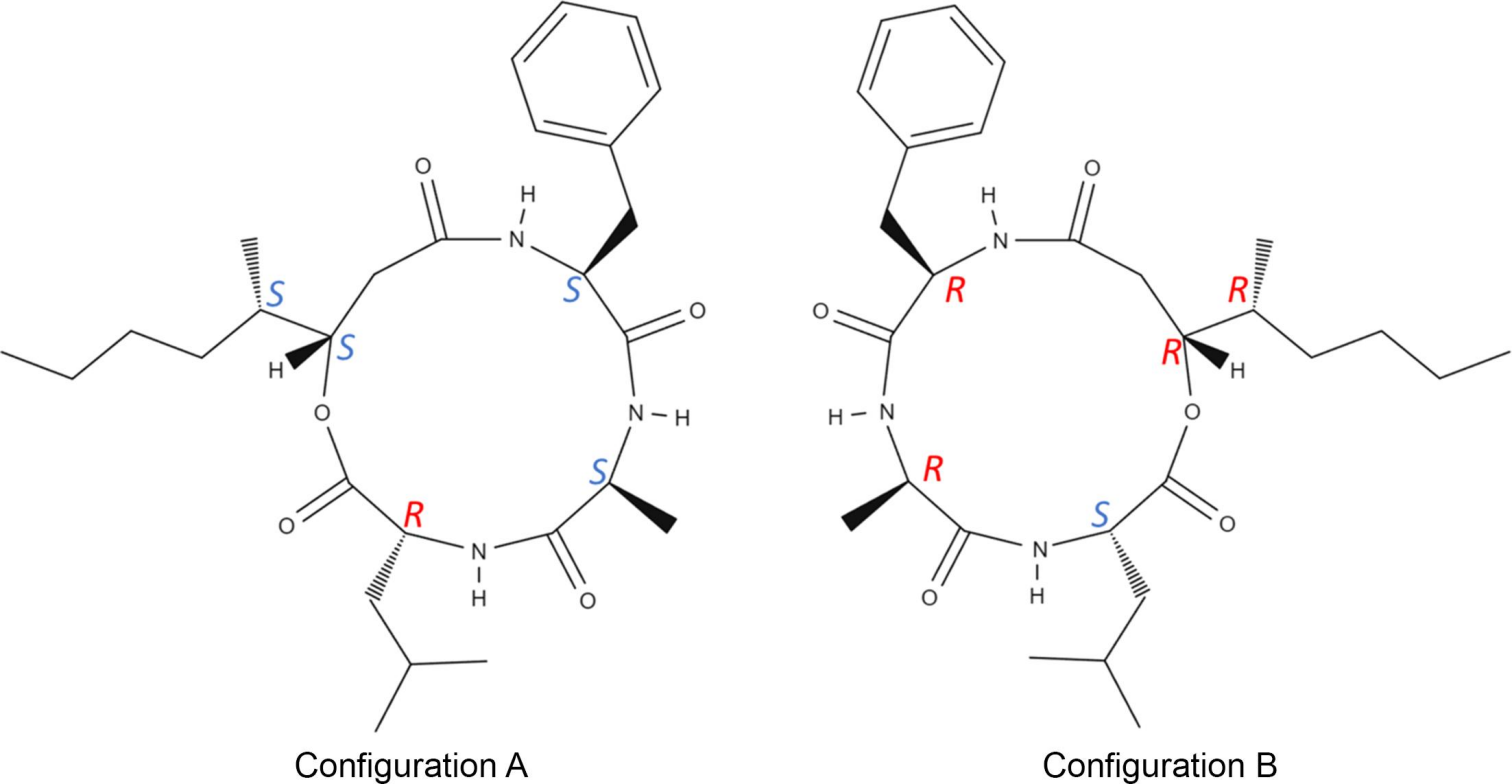
Enanti­omers of beauveriolide I labelled ‘Configuration A’ and ‘Configuration B’ for simplicity. For each chiral C atom, their respective *R* and *S* configuration is labelled.

Beauveriolide I is a chiral mol­ecule and has two different enanti­omers (Fig. 6 ▸), which can thus form two enanti­omorphs in crystalline form. Structure models of both enanti­omorphs were refined against the same data set using the same set of parameters and restraints. The correct absolute structure and configuration can be determined easily by comparing the refinement *R* factors (Table 2 ▸). The difference in the *R* factors is significant enough to point to ‘Configuration A’ as the correct enanti­omer. To qu­antify the reliability of the absolute structure determination, we used the z-score method proposed by Klar *et al.* (2023 ▸). The z-score method provides the confidence level that the hypothesis that the selected configuration is correct. The z-score of 23.0σ for ‘Configuration A’ (Table 2 ▸) corresponds to a probability of correct absolute structure estimation indistinguishably close to 100%.

## Conclusion

The crystal structure of beauveriolide I was solved *ab initio* using diffraction data collected from four crystals using continuous-rotation 3D ED. The compound crystallized in the space group *I*2, with lattice parameters of *a* = 40.2744 (4), *b* = 5.0976 (5), *c* = 27.698 (4) Å and β = 105.729 (6)°. After the dynamical refinement of the solved structure without the H atoms in the amine groups, four out of six H atoms were located in the DESP maps, which were found to be in trigonal planar geometry, and the same case was assumed for the other two. The amine H atoms were found to form hydrogen bonds with the O atoms of adjacent mol­ecules along the *b* axis. The absolute structure was determined using the z-score method at the confidence level of 23.0σ. This study, apart from providing the structure of the studied compound, further highlights the utility of the 3D ED technique for studying structures of complex beam-sensitive organic compounds, including natural products. The robustness of the absolute structure determination is an important feature of the method, which is of foremost importance in the analysis of natural products, where the absolute configuration is often unknown and difficult to determine.

## Supplementary Material

Crystal structure: contains datablock(s) global, I. DOI: 10.1107/S2053229624001359/nh3001sup1.cif

Structure factors: contains datablock(s) I. DOI: 10.1107/S2053229624001359/nh3001Isup2.hkl

Supporting information file. DOI: 10.1107/S2053229624001359/nh3001Isup3.cml

Additional figures. DOI: 10.1107/S2053229624001359/nh3001sup4.pdf

CCDC reference: 2332378

## Figures and Tables

**Table 1:** 3D ED experimental, crystal structure and refinement details

3D ED experimental information		
Collection method	Continuous-rotation data collection from four crystals	
Tilt information	Crystal label	α_min_, α_max_, Δα (°)
	**a**	−34.38, 33.99, 0.30
	**b**	−45.05, 16.06, 0.30
	**c**	−44.43, 32.61, 0.30
	**d**	−28.38, 10.60, 0.30
Exposure time (ms)	1014, 504, 504, 504	
Beam diameter (nm)	960, 2150, 1050, 1050	
Camera length (mm)	1500	
		
Crystal information		
Empirical formula	C_27_H_41_N_3_O_5_	
*Z*, *Z*′	8, 2	
Space group	*I*2	
*a*, *b*, *c* (Å)	40.2744 (4), 5.0976 (5), 27.698 (4)	
α, β, γ (°)	90, 105.729 (6), 90	
*V* (Å^3^)	5473.63	
Apparent mosaicities (°)	0.2765, 0.4080, 0.0598, 0.1323	
Completeness (%)	100	
		
Kinematical refinement		
sin (θ_max_)/λ (Å^−1^)	0.55	
*N*_obs_, *N*_all_	3953, 6836	
Parameters	298	
*R*_obs_, *wR*_obs_ (%)	18.39, 23.55	
*R*_all_, *wR*_all_ (%)	24.58, 25.38	
min[Δ*V*(*r*)], max[Δ*V*(*r*)] (e Å^−1^)	−0.96, 1.00	
		
Dynamical refinement		
sin (θ_max_)/λ (Å^−1^)	0.55	
*N*_obs_, *N*_all_	5888, 14562	
Parameters	365	
*R*_obs_, *wR*_obs_ (%)	11.73, 12.07	
*R*_all_, *wR*_all_ (%)	17.45, 12.82	
min[Δ*V*(*r*)], max[Δ*V*(*r*)] (e Å^−1^)	−0.56, 0.53	

Computer programs: *PETS2* (Palatinus *et al.*, 2019 ▸), *JANA2020* (Petříček *et al.*, 2023 ▸), *SHELXT* (Sheldrick, 2015 ▸) and *VESTA* (Momma & Izumi, 2008 ▸).

**Table 2:** Comparison of *R* factors and z-scores between the two enanti­omorphs The z-scores were calculated assuming ‘Configuration A’ is the correct assignment.

	Configuration A	Configuration B
*R*_obs_, *wR*_obs_ (%)	11.81, 12.16	15.21, 16.30
*R*_all_, *wR*_all_ (%)	17.60, 12.91	20.96, 16.98
z-score from crystal **b** [Fig. 2 ▸(*b*)]	21.2σ	
z-score from crystal **c** [Fig. 2 ▸(*c*)]	10.0σ	
z-score from crystals **b** and **c** combined	23.0σ	
