# Supplementary material for: Structure and absolute configuration of natural fungal product beauveriolide I, isolated from Cordyceps javanica, determined by 3D electron diffraction
Source: Acta Crystallogr C Struct Chem. 2024 Feb 27;80(Pt 3):56–61. doi: 10.1107/S2053229624001359 (PMC10913083; doi:10.1107/S2053229624001359)
Supplement: Supplementary file 4 [file c-80-00056-sup4.pdf]

Acta Cryst C STRUCTURAL CHEMISTRY

Volume 80 (2024)

Supporting information for article:

Structure and absolute configuration of natural fungal product beauveriolide I, isolated from *Cordyceps javanica*, determined by 3D electron diffraction

Kshitij Gurung, Petr Šimek, Alexandr Jegorov and Lukáš Palatinus

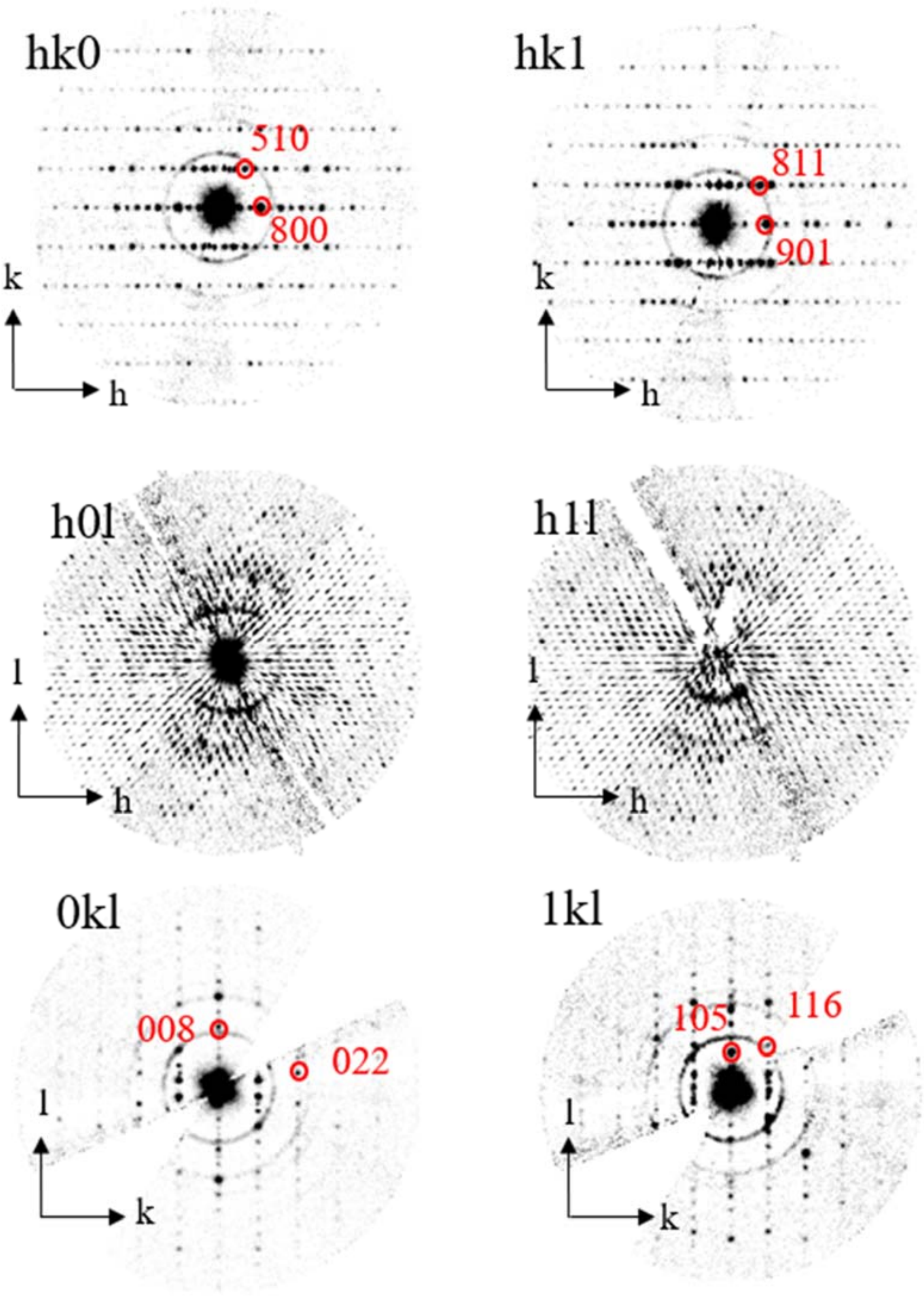

Figure S1 2D reconstruction of the reciprocal sections of beauveriolide I from 3D electron diffraction pattern. The presence of only the reflection condition $h + k + l = 2n$ denotes the presence of I-centering, and hence the space group *I*2.

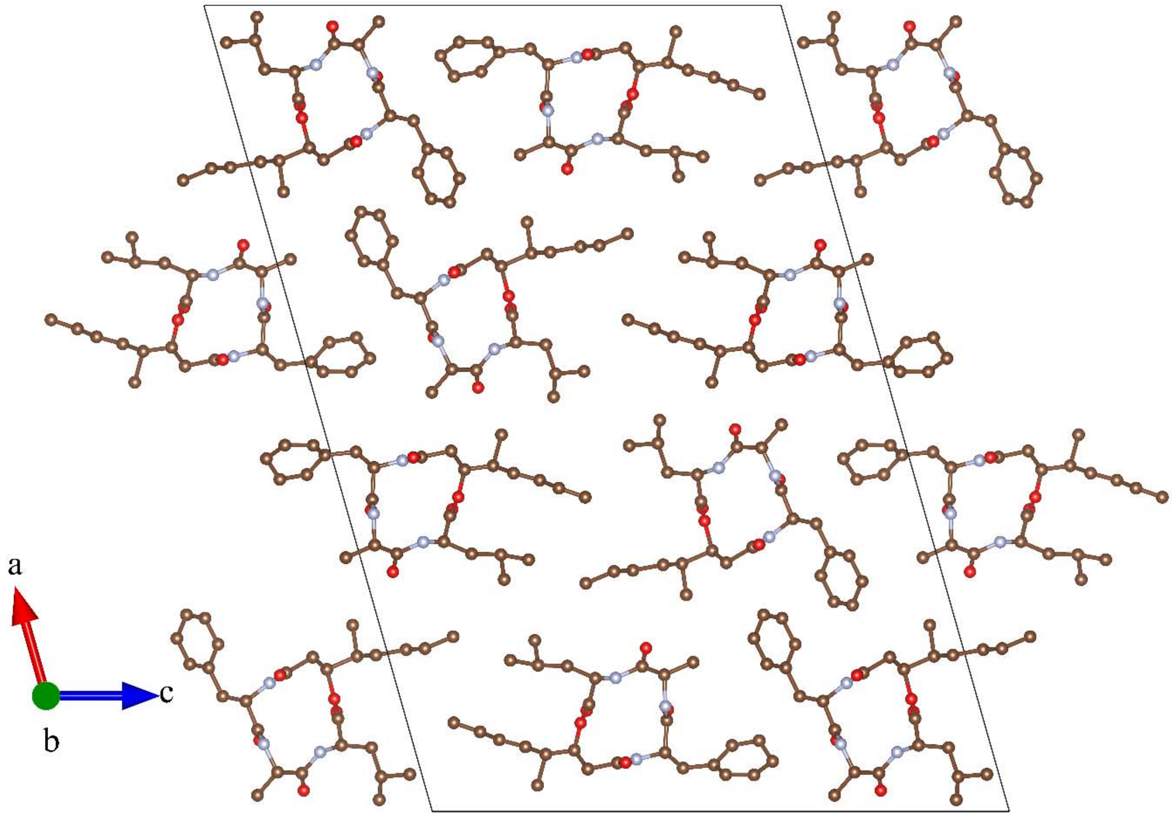

Figure S2 Solved crystal structure of beauveriolide I, viewed down the *c*-axis. Hydrogen atoms were removed for clarity.
